# SEFA: Semantic Embedding-Based Feature Augmentation of Biomedical Language-Model Embeddings Improves Interpretable Metabolomic Prediction of Lung Cancer

**DOI:** 10.3390/biomedicines14081856

**Published:** 2026-08-18

**Authors:** Jiawen Wu, Jean-François Haince, Rashid A. Bux, Guoyu Huang, Paramjit S. Tappia, Bram Ramjiawan, Maria Vaida

**Affiliations:** 1 Department of Data Science, Harrisburg University of Science and Technology, Harrisburg, PA 17101, USA; jwu3@my.harrisburgu.edu; 2 BioMark Diagnostic Solutions Inc., Quebec, QC G1P 4P5, Canada; jhaince@biomarkdiagnostics.com (J.-F.H.); ghuang@biomarkdiagnostics.com (G.H.); 3 BioMark Diagnostics Inc., Richmond, BC V6X 2W2, Canada; rahmed@biomarkdiagnostics.com; 4 Asper Clinical Research Institute and Albrechtsen Research Centre, St. Boniface Hospital, Winnipeg, MB R2H 2A6, Canada; ptappia@sbrc.ca (P.S.T.); bramjiawan@sbrc.ca (B.R.); 5 Department of Pharmacology & Therapeutics, Max Rady College of Medicine, University of Manitoba, Winnipeg, MB R3E 0T6, Canada

**Keywords:** lung cancer, metabolomics, feature augmentation, biomedical language models, semantic embeddings, pathway enrichment, interpretable machine learning, HMDB

## Abstract

**Background/Objectives**: Feature engineering remains a major challenge in metabolomics-based prediction, particularly when rich biochemical knowledge is available but underutilized. Conventional metabolomics models rely primarily on measured variables and statistically driven feature selection, overlooking the molecular and pathway context encoded in curated metabolite knowledge bases. We propose SEFA (Semantic Embedding-based Feature Augmentation), a model-agnostic framework that integrates metabolite-level textual knowledge from the Human Metabolome Database (HMDB) into structured metabolomics modeling for lung-cancer prediction. **Methods**: SEFA encodes HMDB metabolite descriptions as 768-dimensional MedBERT vectors and projects measured metabolite concentrations into this semantic space via concentration-weighted aggregation, producing a 928-dimensional candidate feature matrix that concatenates 11 clinical variables, 149 metabolite concentrations, and 768 semantic projection features. Sparse L1-guided feature selection reduced this representation to 24 features (2.6% of candidates) within a leakage-free cross-validation pipeline. Six classifiers were evaluated on a lung-cancer plasma metabolomics cohort of 800 participants (586 cases, 214 controls) with a stratified 80:20 split, and a controlled ablation study compared the augmented representation with a 24-metabolite-only baseline. Pathway-enrichment analysis of the metabolite sets associated with the retained embedding dimensions was performed using MetaboAnalyst 5.0. **Results**: Logistic regression on the 24-feature SEFA representation achieved a test ROC-AUC of 0.969, a precision-recall AUC of 0.987, and an accuracy of 94.4%, competitive with less interpretable approaches. Under the same 24-feature budget, embedding augmentation improved test ROC-AUC by 0.008, precision-recall AUC by 0.004, and accuracy by 3.1 percentage points over the metabolite-only baseline. Five retained embedding dimensions mapped onto coherent metabolic themes—sphingolipids and acylcarnitines, carnitine and glutamine metabolism, one-carbon and nitrogen handling, purine catabolism, and oxidative stress markers—and their associated metabolite sets were enriched for arginine and proline metabolism and glycine, serine, and threonine metabolism, pathways with established roles in lung-cancer biology. **Conclusions**: SEFA demonstrates that semantic embeddings derived from biomedical language models can convert curated metabolite annotations into patient-level features that supply complementary predictive signal while preserving biological interpretability through pathway-level analysis. The present evidence is limited to a single region-specific cohort; external, cross-platform, and cross-disease evaluation is required before broader generalization or clinical application.

## 1. Introduction

Lung cancer remains the leading cause of cancer mortality worldwide, and outcomes depend strongly on the stage at diagnosis, motivating the search for accurate, minimally invasive biomarkers [1]. Metabolomics, which measures small-molecule end-products of cellular activity, offers a sensitive readout of the metabolic reprogramming that accompanies malignancy and has shown promise for non-invasive lung-cancer detection from blood and other biofluids [2,3,4,5,6,7,8,9].

Most metabolomic classifiers, however, operate purely on the measured concentrations of a metabolite panel and treat each feature as an anonymous number. This discards the substantial prior knowledge that exists for each metabolite in curated resources such as the Human Metabolome Database (HMDB) [10,11], where free-text descriptions summarize biochemical roles, pathway membership, tissue localization, and disease associations. Recent biomedical language models trained on large corpora of clinical and biochemical text encode this knowledge in dense vector representations, and such embeddings have proven transferable across downstream tasks [12,13,14,15,16,17,18,19,20,21,22,23,24,25].

**Figure 1:**
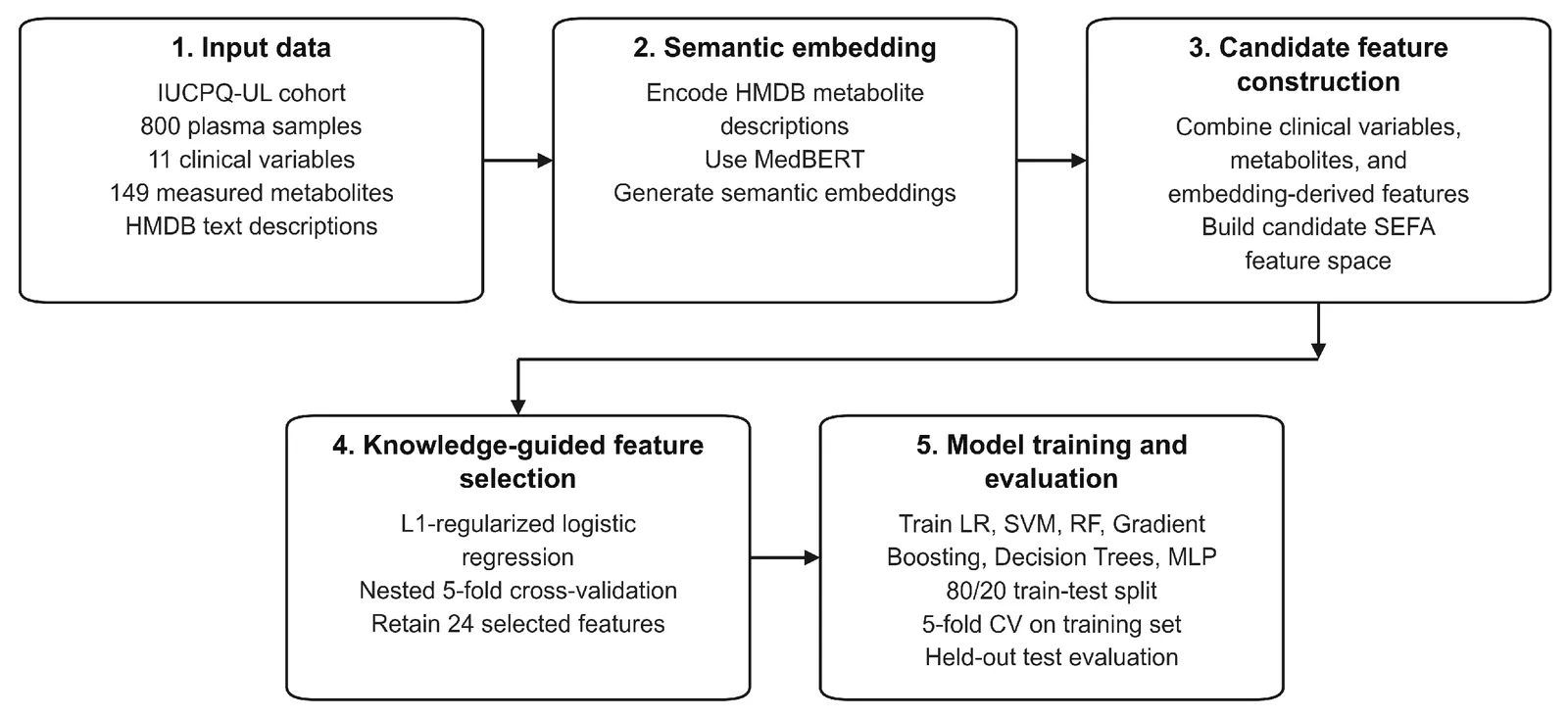
Overview of the SEFA pipeline. Clinical variables, measured metabolite concentrations, and patient-level semantic projections derived from HMDB descriptions represent input features. Sparse feature selection and classifier tuning are performed using nested cross-validation, followed by a single evaluation on the held-out test set.

Here we ask whether transferring this textual, knowledge-derived signal into a metabolomic model improves prediction without sacrificing interpretability. We introduce *SEFA*, which concatenates measured metabolite features with semantic projections constructed from HMDB descriptions and then performs sparse selection within the training data. SEFA provides a model-agnostic bridge from metabolite-level text to sample-level numerical features. This differs from studies that use embeddings only for text classification or static similarity analysis. We benchmark SEFA on a lung-cancer metabolomics cohort, quantify the gain attributable to semantic augmentation through ablation, and interpret the selected embedding dimensions through metabolite loadings and pathway-enrichment analysis. An overview of SEFA workflow is shown in Figure 1.

## 2. Materials and Methods

### 2.1. Dataset and Cohort Characteristics

The study used 800 plasma samples from the IUCPQ Biobank–Respiratory Health Research Network, comprising 586 lung-cancer cases and 214 controls. Samples and associated clinical data were obtained under the University of Manitoba Health Research Ethics Board approval (Ethics File H2012:334, approved 12 December 2022), and all participants provided informed consent. The cohort is therefore region-specific and should not be interpreted as representative of all geographic or ancestry groups. A stratified 80:20 split produced 640 development samples and 160 held-out test samples. The test set contained 118 cases and 42 controls.

Each sample included 11 clinical variables (sex, race, smoking status, past smoking, current smoking, age, height, weight, body-mass index, and two pack-year exposure measures) and metabolite measurements. The cohort was approximately balanced by sex (male: n=393, 49.1% female: n=407, 50.9%), and the distribution by disease status is given in Table 1. Missing values were replaced with the assay limits of detection where available. Columns with more than 30% missingness were excluded. Feature scaling was fitted on the training set only; the held-out test set was transformed using the training-set statistics and contributed to no fitting step. The participant-level dataset cannot be publicly released because of consent and privacy restrictions.

### 2.2. Semantic Embeddings of Metabolite Descriptions

For each metabolite with an available annotation, the curated HMDB textual description was matched to the corresponding assay variable and encoded as a 768-dimensional MedBERT vector. For each participant, measured concentrations were first transformed as sign(x)log(1+|x|). Each participant’s transformed profile was shifted to be non-negative by subtracting its row minimum and normalized to sum to one, giving a weight vector over metabolites. The 768-dimensional semantic representation was then formed as S=WE, where W is the participant-by-metabolite weight matrix and E the metabolite-by-embedding matrix. Because the weights are normalized, the representation is a convex combination of metabolite embeddings and therefore reflects a participant’s metabolite composition rather than overall concentration magnitude. These semantic features, together with the clinical and log-transformed metabolite features, were standardized using statistics estimated from the train set. The encoder weights were fixed and were not trained on case/control labels.

### 2.3. Feature Construction and Selection

Clinical, metabolite, and semantic features were concatenated into the candidate feature matrix. Sparse selection was restricted to the test data and was placed inside the cross-validation pipeline so that no held-out fold contributed to selector fitting. The L1 regularization strength and the maximum retained feature count were treated as tuning parameters. Candidate feature budgets from 4 to 40 and regularization values from $10^{-3}$ to $10^{1}$ were considered. The compact model retained 24 of 928 candidate variables (2.6%). This size reflects the cross-validated sparsity/performance trade-off, not a biological claim that exactly 24 variables are universally optimal.

### 2.4. Classification, Tuning, and Evaluation

Six classifiers were evaluated, namely logistic regression, support vector machine, random forest, multilayer perceptron, gradient boosting, and decision tree. All preprocessing, feature selection, and hyperparameter tuning operations that depend on sample statistics were fit within training folds. The final model used an L2 penalty with the liblinear solver, regularization strength C=1.0, a maximum of 400 iterations, and random seed 42. Feature selection used an L1-penalized logistic regression (liblinear solver, random seed 0) retaining at most 24 features. Five-fold nested cross-validation optimized ROC-AUC. The held-out test set was evaluated once after model selection. Performance was summarized using ROC-AUC, average precision (PR-AUC), accuracy, and confusion matrix.

The analysis was conducted in Python 3.11 using Pandas, NumPy, SciPy, and Scikit-Learn libraries for data handling and modeling and Matplotlib (v3.10.0) for visualization. The metabolite-text semantic features were generated with the pretrained MedBERT model loaded through the Hugging Face Transformers library. The specific embedding dimensionality is reported in the Materials and Methods. Textual metabolite descriptions are publicly available in HMDB database. Pathway-enrichment significance is reported as $-\log_{10}\left(p\right)$ with false-discovery-rate correction, and an adjusted *p*-value p<0.05 was considered significant.

## 3. Results

### 3.1. Predictive Performance and Model Selection

Across the six classifiers, logistic regression achieved the highest cross-validated ROC-AUC (mean 0.9323, σ=0.0213), narrowly ahead of SVM (0.9320), with random forest, MLP and gradient boosting clustered near 0.92 and decision tree lowest at 0.8187 (Table 2). The same ranking held for PR-AUC and accuracy. Per-fold ROC-AUC and the cross-validation-versus-test comparison indicated that the top models generalized well.

The ablation study in Table 3 isolates the effect of adding embeddings under the same 24-feature count. The 24-metabolite-only logistic regression model achieved a cross-validation ROC-AUC of 0.9219 ± 0.0367, a test ROC-AUC of 0.9609, a test PR-AUC of 0.9832, and a test accuracy of 0.9125. After embedding-derived features were incorporated into the final 24-feature representation, performance gains were observed of 0.0104 in cross-validation ROC-AUC, 0.0082 in test ROC-AUC, 0.0039 in test PR-AUC, and 0.0312 in test accuracy. This gain is statistically and practically smaller than the absolute performance values and should be interpreted as evidence of complementary signal, not as proof of a clinically meaningful advantage. Notably, several selected features were embedding dimensions rather than measured metabolites (e.g., dimensions 368, 495, 514, 609 and 685), indicating that the language-model representation contributed signal that the measured panel did not fully capture. These results show that the embedding features recovered predictive information that would otherwise be lost under aggressive feature reduction, allowing the compact representation to perform modestly better than a metabolite-only model of the same size.

**Figure 2:**
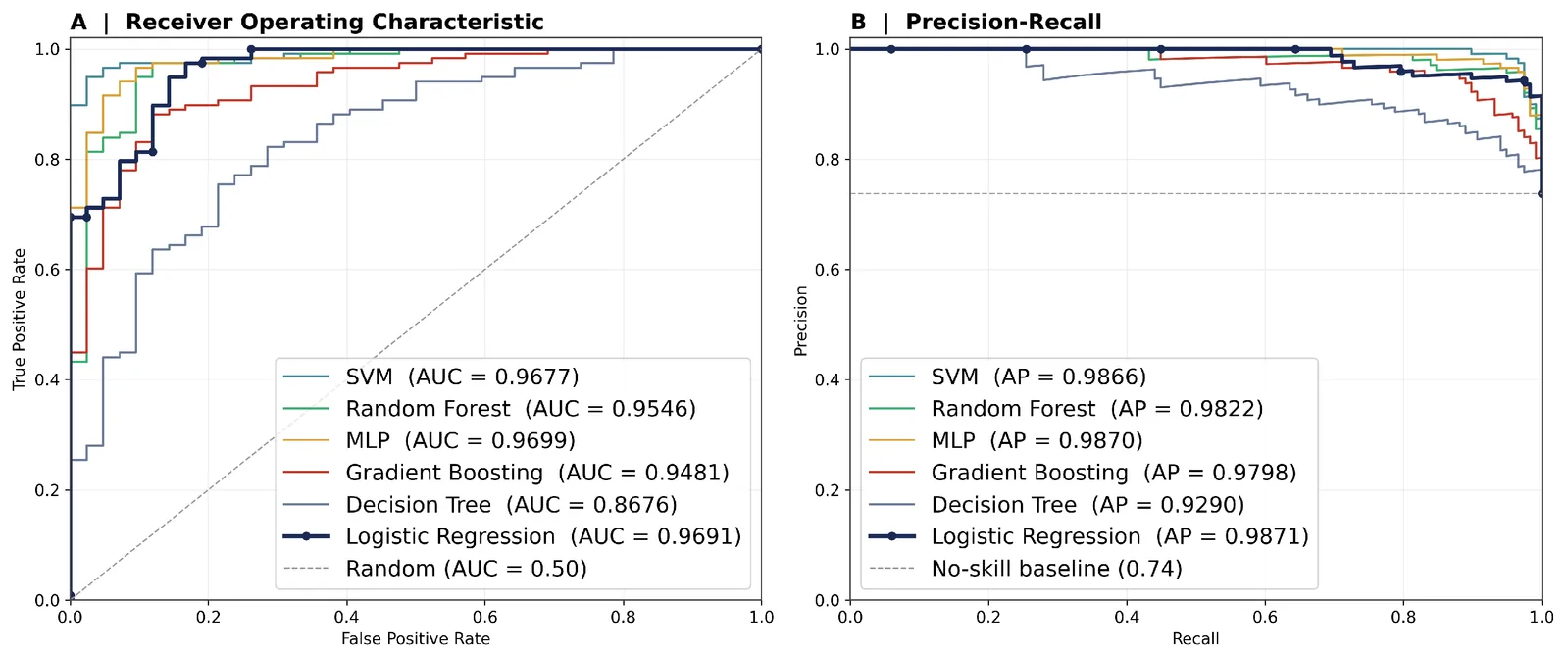
Test-set performance curves for six classifiers trained on the final 24-feature SEFA representation. (**A**) Receiver Operating Characteristic (ROC) curves. Logistic Regression (AUC = 0.9691) and MLP (AUC = 0.9699) achieve the highest area under the curve. (**B**) Precision-Recall (PR) curves. Logistic Regression attains the highest average precision (AP = 0.9871).

**Figure 3:**
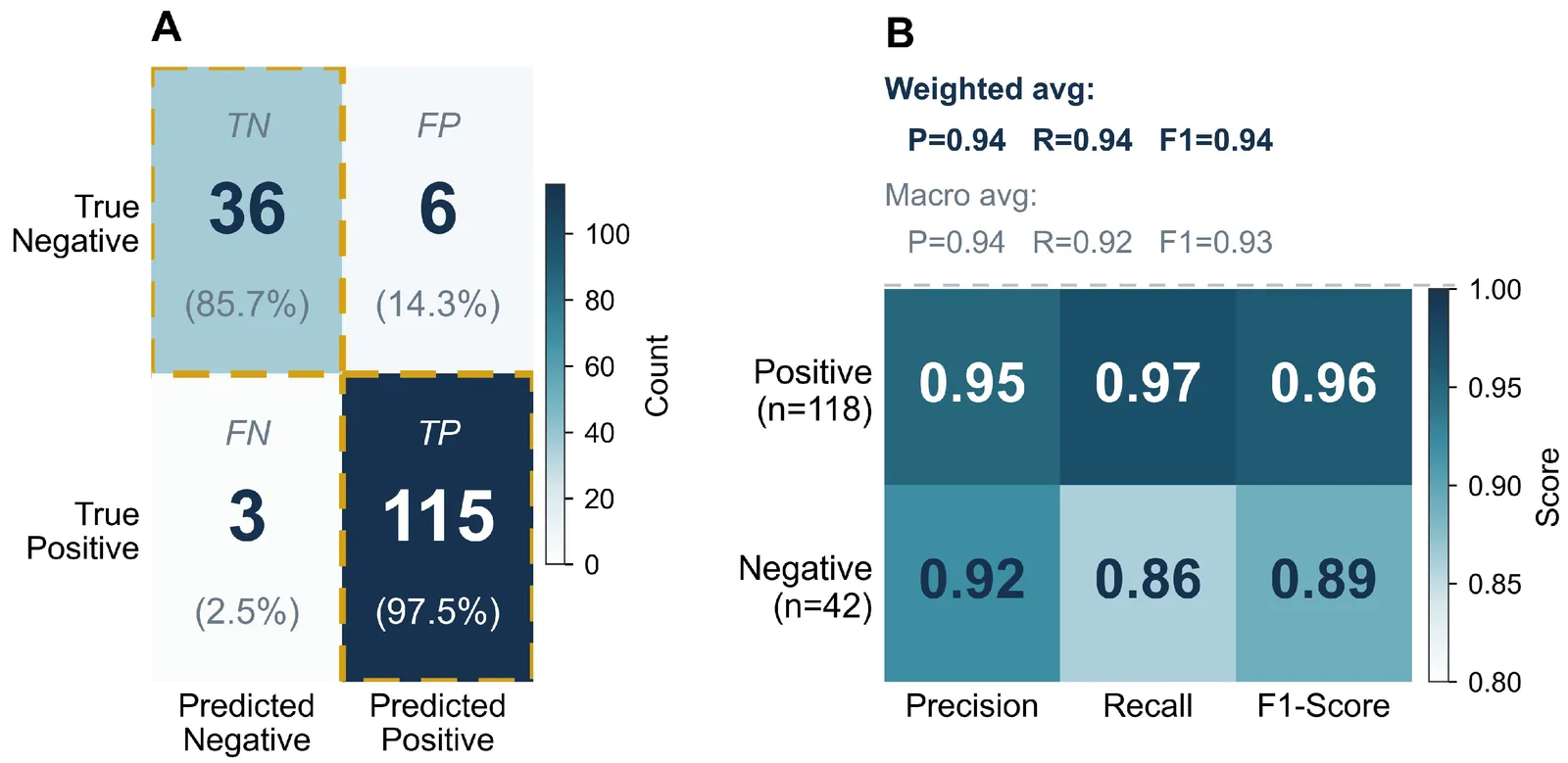
Evaluation of the final logistic regression model on the 24-feature representation. Panel (**A**) shows the confusion matrix on the held-out test set. Panel (**B**) shows the class-specific precision, recall, and F1-score together with macro and weighted averages.

The regularized logistic-regression model selected 24 features and achieved a held-out test ROC-AUC of 0.9691 and a PR-AUC of 0.9871 (Figure 2). The confusion matrix contained 6 false-positive controls and 3 false-negative cancer cases and had strong precision, recall and F1 score on the positive class (Figure 3A,B).

### 3.2. Interpretation of Selected Embedding Dimensions

To understand what the retained embedding dimensions encode, we ranked the metabolites loading most strongly on each (Supplementary Figure S1) and summarized their description vocabulary as word clouds (Supplementary Figure S2). These loading and word-cloud displays are exploratory attribution aids, without claiming any mechanistic or causal results. The dimensions captured lung cancer-associated metabolites. Dimension 368 was dominated by sphingolipids and acylcarnitines [26,27], dimension 495 by carnitine/glutamine-related terms [28], dimension 514 by GABA, sarcosine and creatine-related species [29], dimension 609 by trimethylamine and hypoxanthine [30], and dimension 685 by urate, spermine and methionine-related chemistry [31,32,33].

### 3.3. Pathway Enrichment of Embedding-Derived Metabolite Sets

**Figure 4:**
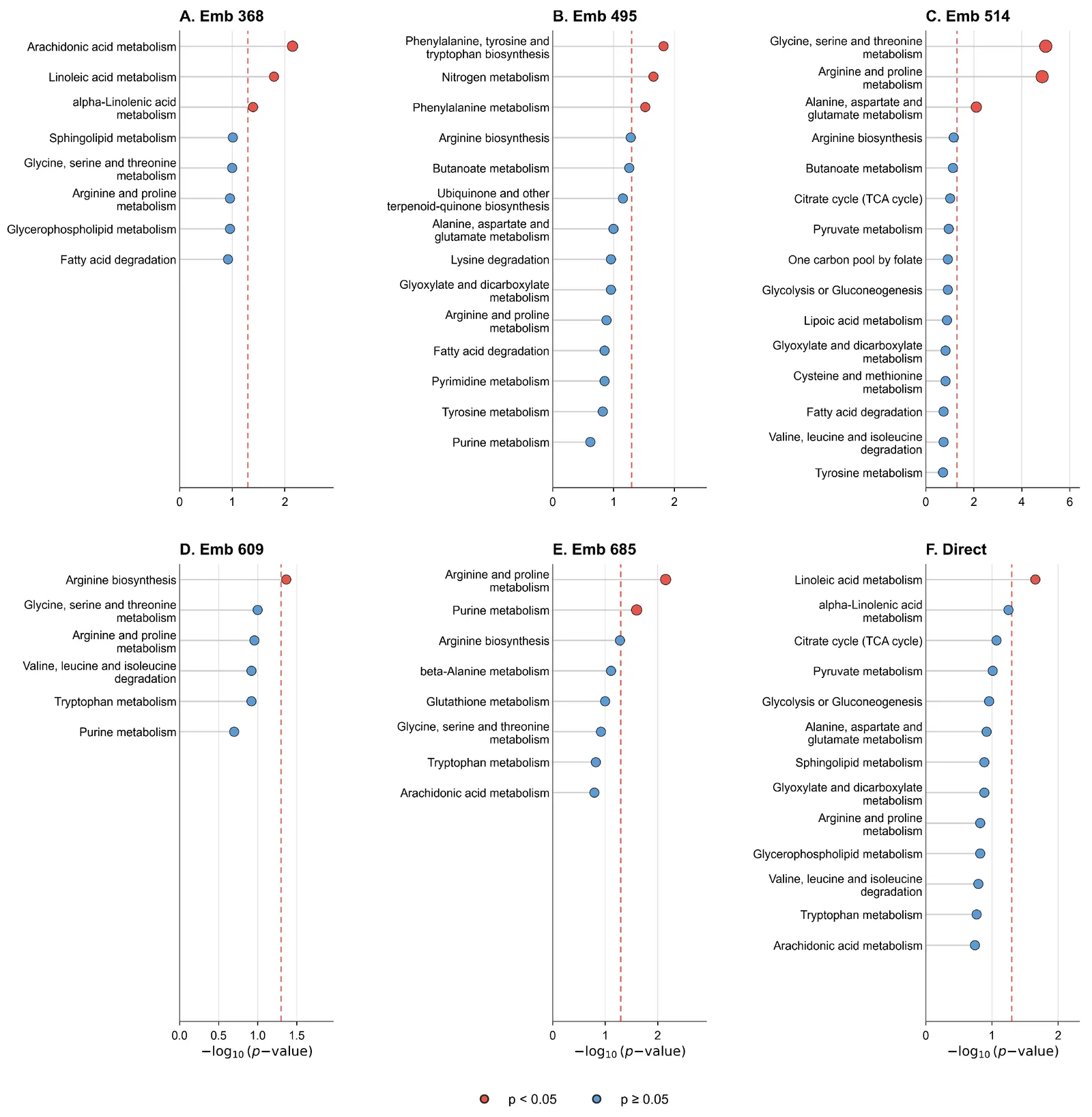
Metabolic pathway enrichment for each feature source. Panels (**A**–**F**) correspond to the five selected MedBERT embedding dimensions and the directly selected metabolites. Each row is an enriched KEGG pathway, positioned by −log_10_(*p*) from Fisher’s exact test against the 149-metabolite background. The red dashed line marks p=0.05 ($-\log_{10}p=1.30$). Points to its right are enriched at nominal significance.

**Figure 5:**
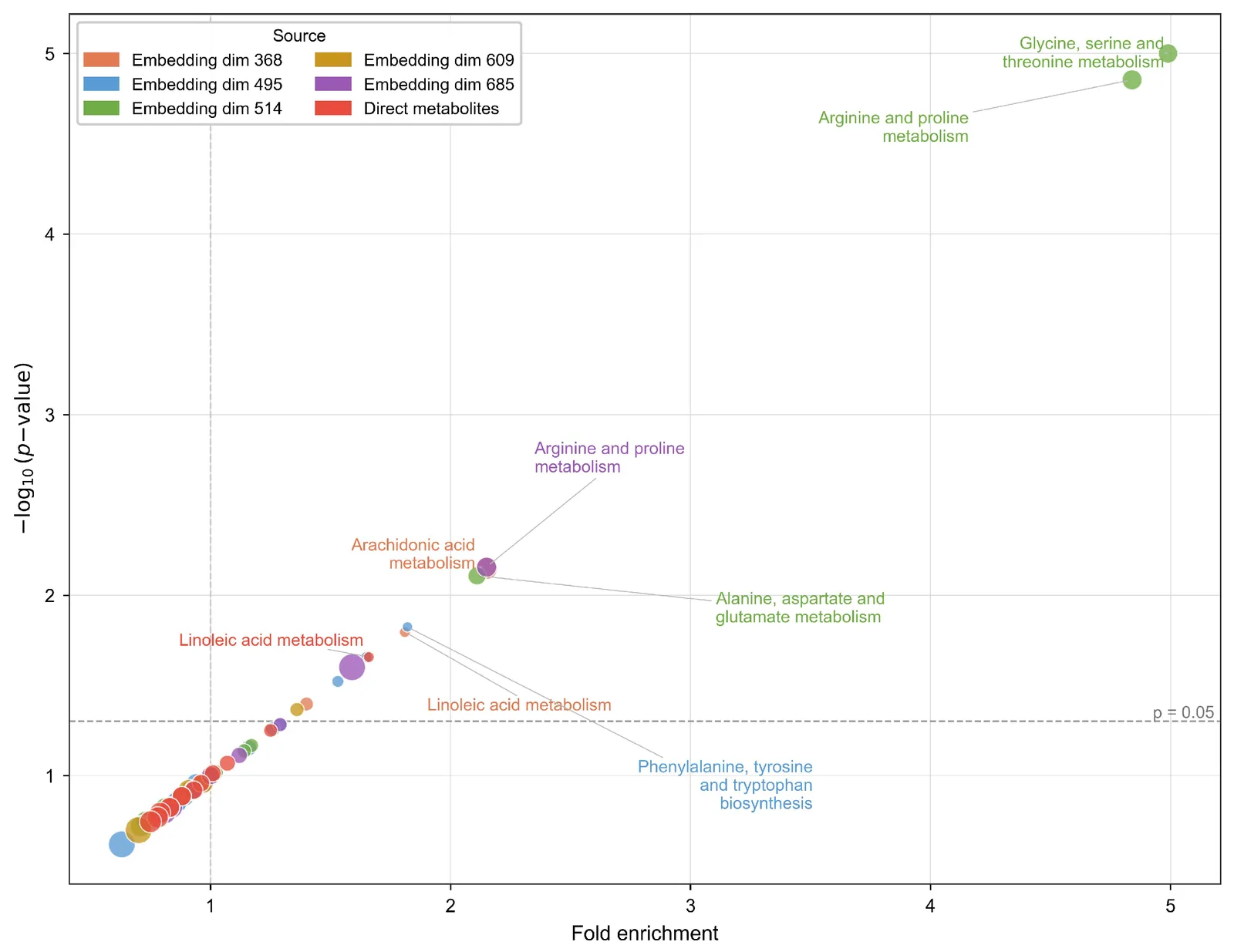
Combined significance-versus-enrichment scatter across all sources. Each point is a pathway colored by its source. The dashed line marks p=0.05. Glycine, serine and threonine metabolism and arginine and proline metabolism (embedding 514) emerge as the most significant, high-fold-enrichment pathways.

**Figure 6:**
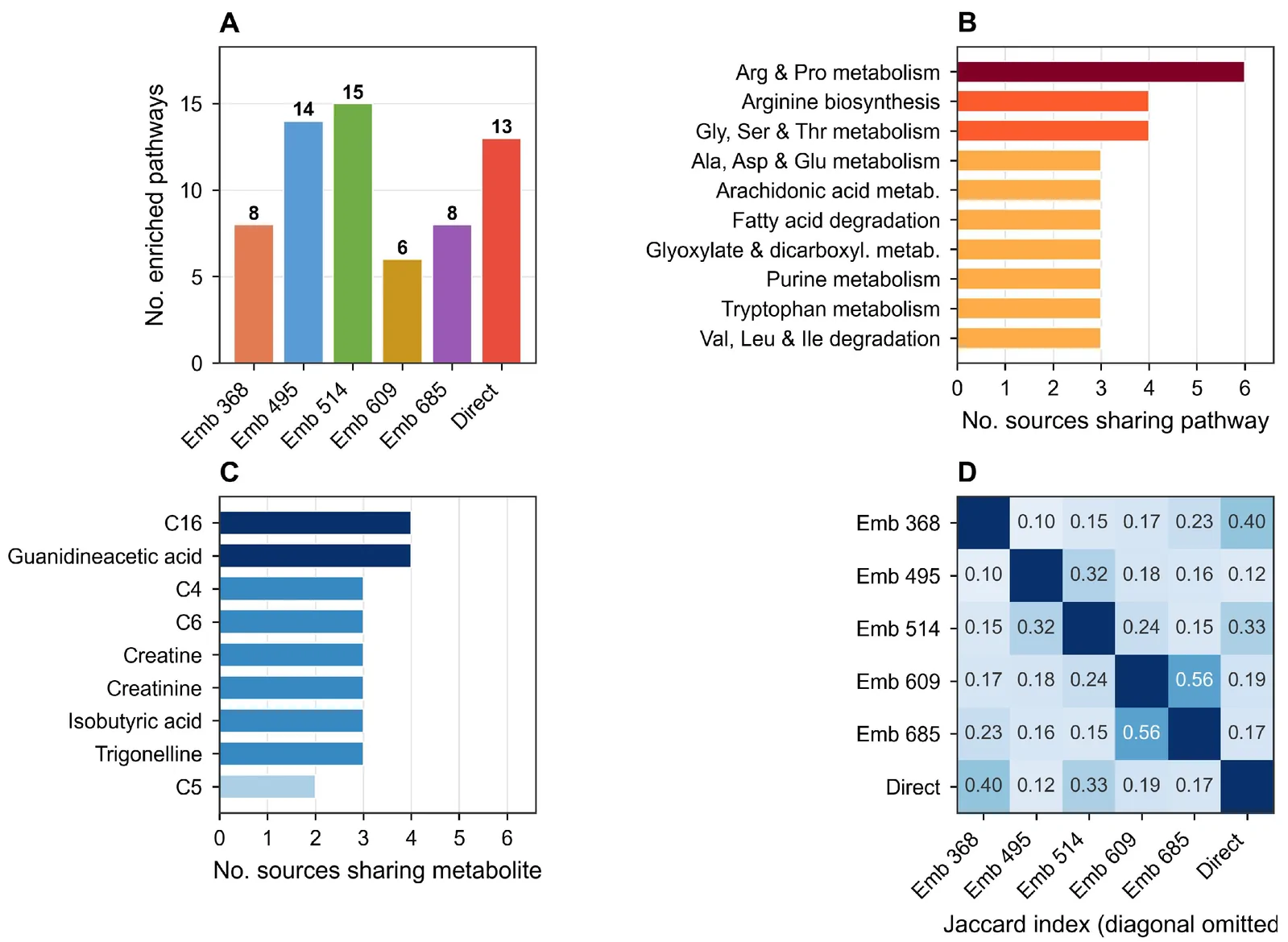
Cross-source comparison. (**A**) Number of enriched pathways per source. (**B**) Pathways ranked by the number of sources sharing them. (**C**) Metabolites ranked by sharing across sources. (**D**) Pairwise Jaccard similarity between sources, showing that embedding dimensions capture partly distinct pathway sets.

Pathway-enrichment analysis of the metabolite sets associated with each embedding dimension, compared against direct metabolite selection, recovered lung-cancer-relevant biology (Figure 4 and Figure 5). Arginine and proline metabolism was the most broadly shared pathway, appearing across all six sources, followed by arginine biosynthesis and glycine, serine and threonine metabolism (Figure 6B). Embedding dimension 514 produced the strongest enrichment signals—glycine, serine and threonine metabolism (∼5-fold) and arginine and proline metabolism (∼4.8-fold), exceeding those obtained from direct metabolites.

The number of significantly enriched pathways varied by source (15 for embedding 514, 14 for 495, 13 for direct metabolites, and 6–8 for the remaining dimensions. Figure 6A), and pairwise Jaccard similarity showed that different embedding dimensions captured partly non-overlapping pathway sets (Figure 6D), consistent with the embeddings supplying complementary information.

## 4. Discussion

SEFA shows that semantic embeddings of biomedical text can be folded into a metabolomic classifier as additional features and that they yield an improvement in lung-cancer prediction (+0.008 ROC-AUC, +0.031 accuracy) while keeping the final model compact and interpretable. This gain should be interpreted as evidence of a complementary signal, not as proof of a clinically meaningful advantage. The best model—an L2-regularized logistic regression using only 24 of 928 candidate features—reached a test ROC-AUC of 0.969 and 94.4% accuracy, performance competitive with far less transparent approaches.

The selected latent dimensions mapped onto coherent metabolite groups (sphingolipids, acylcarnitines, carnitine/glutamine chemistry, GABA/creatine species), and their metabolite sets were enriched for pathways with established links to malignant metabolic reprogramming, most consistently arginine and proline metabolism and glycine, serine, and threonine metabolism. The partly non-overlapping pathway coverage across dimensions (low pairwise Jaccard similarity) supports the view that the embeddings contribute complementary information to the measured panel, which is consistent with the ablation result.

The pathways most consistently recovered have established links to lung-cancer biology. Arginine and proline metabolism is implicated in lung adenocarcinoma prognosis and immune escape, and proline catabolism has been shown to drive progression in non-small-cell lung cancer, consistent with the reduced arginine availability reported in cancer more broadly [34,35,36]. Glycine, serine and threonine metabolism feed one-carbon metabolism, a recognized hallmark of proliferating tumors [37], while purine metabolism has been linked to macrophage-mediated immunosuppression in non-small-cell lung cancer [38]. These convergent signals, together with the broader lung-cancer metabolomic disturbances reported previously [7,8,39,40], support the biological plausibility of the embedding-derived features.

This study is subject to several limitations. All participants were drawn from a single biobank network, and the cohort was imbalanced toward cancer cases. Differences in geography, ancestry, sex, clinical stage, and acquisition protocol may therefore constrain the transportability of the model. The held-out partition provides an estimate of internal generalization only and cannot substitute for temporal, cross-site, or prospective validation. The semantic features are contingent on the completeness, phrasing, and update cycle of the HMDB annotations, as well as on the choice of language model and the metabolite-name mapping procedure. In addition, both the selected feature-set size and the specific variables retained may vary across resampling iterations, indicating a degree of selection instability. The pathway-enrichment results and embedding loadings are associative and do not establish causal mechanisms. Finally, the observed augmentation gain is modest and may not warrant the additional implementation complexity in all settings.

Accordingly, SEFA should presently be viewed as a research framework rather than a deployable diagnostic system. Future work will evaluate it on independent cohorts, other cancer and non-cancer metabolomics datasets, different assay platforms, and prospectively collected samples. Such studies should include subgroup performance, calibration, decision-curve analysis, and prespecified clinical thresholds before any claim of clinical utility.

## 5. Conclusions

SEFA demonstrates that biomedical language-model embeddings of metabolite descriptions can be converted into patient-level features that supply biologically coherent and statistically complementary information to measured metabolite concentrations and clinical variables. Within this single-cohort study, the augmented representation improved lung-cancer prediction modestly but consistently, and the retained embedding dimensions captured known hallmarks of malignant metabolic reprogramming through pathway-enrichment analysis. SEFA should be regarded as a research framework whose clinical utility, transportability, and scalability require independent evaluation. The results nonetheless position semantic knowledge augmentation as a principled and reproducible strategy for enriching metabolomics-based predictive models with biochemical domain knowledge.

## Figures and Tables

**Table 1:** Demographic and clinical characteristics of the study cohort, stratified by disease status.

Characteristic	Overall	Control	Cancer	*p*	*Q* (BH-FDR)	SMD
	(*n* = 800)	(*n* = 214)	(*n* = 586)			
Sex, *n* (%)	800			0.097	0.117	0.139
Female	407 (50.9)	98 (45.8)	309 (52.7)			
Male	393 (49.1)	116 (54.2)	277 (47.3)			
Race/ethnicity, *n* (%)	800			<0.001	<0.001	0.455
Other	78 (9.8)	3 (1.4)	75 (12.8)			
White	722 (90.2)	211 (98.6)	511 (87.2)			
Smoking status, *n* (%)	800			<0.001	<0.001	0.974
Never smoker	140 (17.5)	98 (45.8)	42 (7.2)			
Ever smoker	660 (82.5)	116 (54.2)	544 (92.8)			
Former smoker, *n* (%)	800			<0.001	<0.001	0.599
No	304 (38.0)	126 (58.9)	178 (30.4)			
Yes	496 (62.0)	88 (41.1)	408 (69.6)			
Current smoker, *n* (%)	800			0.002	0.003	0.265
No	636 (79.5)	186 (86.9)	450 (76.8)			
Yes	164 (20.5)	28 (13.1)	136 (23.2)			
Smoking history, *n* (%)	800			<0.001	<0.001	0.599
No	304 (38.0)	126 (58.9)	178 (30.4)			
Yes	496 (62.0)	88 (41.1)	408 (69.6)			
Age, years	64.00 [58.00–70.00]	60.10 [53.00–68.75]	65.00 [60.00–70.00]	<0.001	<0.001	0.381
Height, m	1.65 [1.58–1.72]	1.68 [1.60–1.73]	1.64 [1.57–1.70]	<0.001	<0.001	−0.322
Weight, kg	73.00 [62.00–84.00]	74.00 [63.50–86.00]	72.00 [61.00–84.00]	0.198	0.198	−0.085
Body mass index	26.67 [23.53–30.09]	26.03 [23.67–29.17]	26.84 [23.52–30.37]	0.160	0.174	0.127
Smoking exposure, pack-years	30.30 [10.00–48.00]	3.50 [0.00–25.00]	40.00 [21.38–50.00]	<0.001	<0.001	1.032
Smoking exposure, cigarettes/day	20.00 [7.00–20.00]	0.00 [0.00–15.00]	20.00 [10.00–20.00]	<0.001	<0.001	0.879

Note: Continuous variables are summarized as median [interquartile range] or mean ± SD according to the Shapiro–Wilk test for normality; categorical variables as *n* (%), with percentages computed within column. *p* values are two-sided, from the test named in each row; *Q* values are Benjamini–Hochberg false-discovery-rate–adjusted across all variables in the table. SMD, standardized mean difference; |SMD|<0.10 indicates negligible imbalance.

**Table 2:** Classifier comparison on the final 24-feature representation. Bold values indicate the best result in each metric. CV metrics are the mean over 5-fold cross-validation (±standard deviation for ROC-AUC); test metrics are computed once on the held-out set (n=160).

Model	CV ROC-AUC	CV F1	Test ROC-AUC	Test PR-AUC	Acc.
Logistic Regression	**0.9323 ± 0.0213**	0.9242	0.9691	**0.9871**	**0.9437**
SVM	0.9320 ± 0.0174	0.9202	0.9677	0.9866	0.9187
Random Forest	0.9214 ± 0.0101	0.9201	0.9546	0.9822	0.9000
MLP	0.9213 ± 0.0192	0.9172	**0.9699**	0.9870	0.9375
Gradient Boosting	0.9201 ± 0.0115	**0.9236**	0.9481	0.9798	0.9125
Decision Tree	0.8187 ± 0.0392	0.8667	0.8676	0.9290	0.8375

**Table 3:** Ablation study for logistic regression under a fixed 24-feature budget. The proposed 24-feature representation with embeddings is compared with a 24-metabolite-only baseline.

Feature Set	CV ROC-AUC	Test ROC-AUC	Test PR-AUC	Acc.
24 metabolites only	0.9219 ± 0.0367	0.9609	0.9832	0.9125
24 features + embeddings	0.9323 ± 0.0213	0.9691	0.9871	0.9437
Δ (with emb. − met. only)	+0.0104	+0.0082	+0.0039	+0.0312

## Data Availability

Participant-level data are not publicly available because of privacy, consent, and institutional restrictions. A de-identified data dictionary and access requests may be directed to the corresponding author and data custodian, subject to ethics and institutional approval. All code implementing the SEFA method is publicly available at https://github.com/miliana/SEFA, accessed on 28 July 2026. The repository provides a complete, self-contained reference implementation of semantic embedding feature augmentation, including construction of the abundance-weighted embedding, the L1-penalized feature selection, and the cross-validation and held-out evaluation procedure. It runs on synthetic data generated by the accompanying script, so the pipeline can be executed and inspected without access to participant-level data.

## Supplementary Materials

The following supporting information can be downloaded at: https://www.mdpi.com/article/10.3390/biomedicines14081856/s1, Figure S1: Top 10 metabolites per selected embedding dimension, ranked by cosine-similarity loading. Each panel lists the metabolites whose HMDB-description embeddings align most strongly with embedding dimensions 368, 495, 514, 609 and 685. These loadings are exploratory attribution aids that suggest the chemical themes associated with each latent dimension. Figure S2: Word clouds of HMDB description vocabulary associated with each selected embedding dimension (368, 495, 514, 609, 685). Term prominence reflects frequency within the descriptions of the top-loading metabolites.

## Abbreviations

The following abbreviations are used in this manuscript:

SEFA	Semantic Embedding-Based Feature Augmentation
HMDB	Human Metabolome Database
ROC-AUC	Area Under the Receiver Operating Characteristic Curve
PR-AUC	Area Under the Precision-Recall Curve (Average Precision)
CV	Cross-Validation
FDR	False Discovery Rate
MLP	Multilayer Perceptron
SVM	Support Vector Machine
TP/TN/FP/FN	True/False Positives and Negatives
