# Supplementary material for: SEFA: Semantic Embedding-Based Feature Augmentation of Biomedical Language-Model Embeddings Improves Interpretable Metabolomic Prediction of Lung Cancer
Source: Biomedicines. 2026 Aug 18;14(8):1856. doi: 10.3390/biomedicines14081856 (PMC13509573; doi:10.3390/biomedicines14081856)
Supplement: Supplementary file 1 [file biomedicines-14-01856-s001.zip › biomedicines-4418554-supplementary.pdf]

MDPI

Article

SEFA: Semantic Embedding-Based Feature Augmentation of Biomedical Language-Model Embeddings Improves Interpretable Metabolomic Prediction of Lung Cancer

Jiawen Wu [1], Jean-Françoise Haince [2], Rashid A. Bux [3], Guoyu Huang [2], Paramjit S. Tappia [4], Bram Ramjiawan [4,5], and Maria Vaida [1,*]

1 Harrisburg University of Science and Technology, Harrisburg, PA, USA; JWu3@my.harrisburgu.edu (J.W.); MVaida@harrisburgu.edu (M.V.)
2 BioMark Diagnostic Solutions Inc., Quebec, QC, Canada; jhaince@biomarkdiagnostics.com (J.-F.H.); ghuang@biomarkdiagnostics.com (G.H.)
3 BioMark Diagnostics Inc., Richmond, BC, Canada; rahmed@biomarkdiagnostics.com
4 Asper Clinical Research Institute and Albrechtsen Research Centre, St. Boniface Hospital, Winnipeg, MB, Canada; ptappia@sbrc.ca (P.S.T.); bramjiawan@sbrc.ca (B.R.)
5 Department of Pharmacology & Therapeutics, Max Rady College of Medicine, University of Manitoba, Winnipeg, MB, Canada
* Correspondence: mvaida@harrisburgu.edu

Supplementary Materials

The following supporting figures present exploratory embedding-dimension attributions. They are qualitative aids for interpreting the retained latent dimensions and do not establish mechanism or causal relevance.

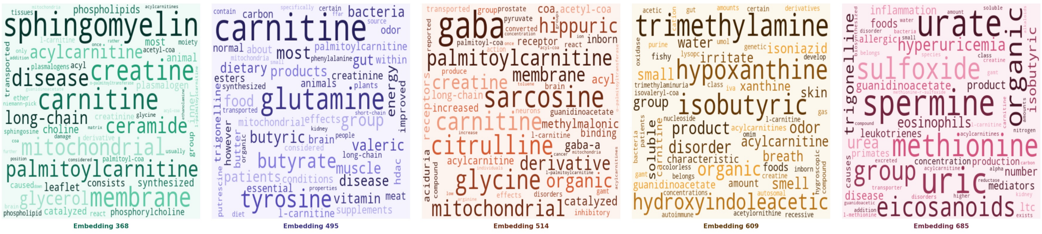

Figure S1. Top 10 metabolites per selected embedding dimension, ranked by cosine-similarity loading. Each panel lists the metabolites whose HMDB-description embeddings align most strongly with embedding dimensions 368, 495, 514, 609 and 685. These loadings are exploratory attribution aids that suggest the chemical themes associated with each latent dimension.

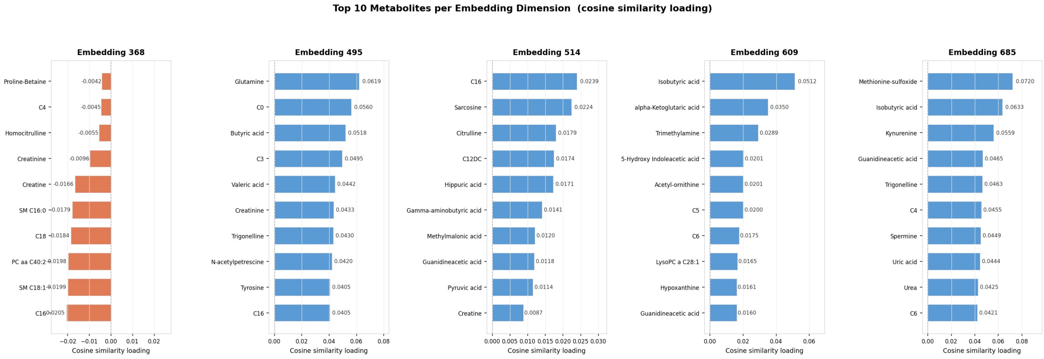

Figure S2. Word clouds of HMDB description vocabulary associated with each selected embedding dimension (368, 495, 514, 609, 685). Term prominence reflects frequency within the descriptions of the top-loading metabolites.
